# Comparative Study of *Clostridium difficile* Clinical Detection Methods in Patients with Diarrhoea

**DOI:** 10.1155/2020/8753284

**Published:** 2020-01-21

**Authors:** Yanyan Xiao, Yong Liu, Xiaosong Qin

**Affiliations:** Department of Clinical Laboratory, Shengjing Hospital of China Medical University, Shenyang, China

## Abstract

**Objectives:**

The aim of this study was to evaluate the clinical application of three methods for detecting *Clostridium difficile* in fecal samples.

**Methods:**

One hundred and fifty fecal specimens were collected and tested for *C. difficile* using three methods: (1) the toxigenic culture (TC); (2) the VIDAS enzyme immunoassay (EIA): the VIDAS glutamate dehydrogenase (GDH) assay and toxin A/B assay were used to detect GDH antigen and A/B toxin; and (3) the GeneXpert PCR assay. The toxigenic culture was used as a reference to evaluate the performance of the VIDAS EIA and the GeneXpert PCR assay.

**Results:**

Of 150 specimens, 26 carried both A and B toxin genes, and none of the samples were positive for the binary toxin gene. Toxin-producing *C. difficile* using three methods: (1) the toxigenic culture (TC); (2) the VIDAS enzyme immunoassay (EIA): the VIDAS glutamate dehydrogenase (GDH) assay and toxin A/B assay were used to detect GDH antigen and A/B toxin; and (3) the GeneXpert PCR assay. The toxigenic culture was used as a reference to evaluate the performance of the VIDAS EIA and the GeneXpert PCR assay. *C. difficile* using three methods: (1) the toxigenic culture (TC); (2) the VIDAS enzyme immunoassay (EIA): the VIDAS glutamate dehydrogenase (GDH) assay and toxin A/B assay were used to detect GDH antigen and A/B toxin; and (3) the GeneXpert PCR assay. The toxigenic culture was used as a reference to evaluate the performance of the VIDAS EIA and the GeneXpert PCR assay.

**Conclusion:**

The VIDAS GDH assay is useful for initial screening of *C. difficile* using three methods: (1) the toxigenic culture (TC); (2) the VIDAS enzyme immunoassay (EIA): the VIDAS glutamate dehydrogenase (GDH) assay and toxin A/B assay were used to detect GDH antigen and A/B toxin; and (3) the GeneXpert PCR assay. The toxigenic culture was used as a reference to evaluate the performance of the VIDAS EIA and the GeneXpert PCR assay.

## 1. Introduction

*Clostridium difficile* (CD) is widely distributed in the natural environment and in human and animal feces. It is also a common cause of intestinal infection in hospital patients. *C. difficile* spores are highly resistant to general disinfection measures and can be present in the environment for several months [1]. *C. difficile* is one of the main causative agents of antibiotic-associated diarrhea (AAD). Diarrhoea occurs as a result of the secretion of *C. difficile* toxin A, toxin B, and binary toxin. Nontoxigenic *C. difficile* is a nonpathogenic bacterium, belonging to the colonization of bacteria in the body [2]. Recently, a more virulent strain of the bacteria, referred to as restriction endonuclease analysis group BI, North American pulsefield type 1, and PCR ribotype 027 (BI/NAP1/027), has emerged and has been linked to a more severe disease with an increased risk for severe complications and high mortality [3]. In 2012, BI/NAP1/027 was first reported in Beijing, China [4].

The main symptoms of *C. difficile* infection are fever, abdominal pain, diarrhoea, and severe pseudomembranous colitis (PMC). Infection may lead to severe complications such as toxic megacolon and intestinal perforation, which may lead to death [5]. Presently, *C. difficile* detection is carried out in some areas of China, but the methods used for detection differ between areas. Additionally, routine detection of *C. difficile* is not carried out in some areas, which is not conducive to rapid detection of the disease. As a result, missed diagnoses and delays in treatment may occur. Currently, no relevant detection data in this area of study have been published. In this study, we evaluated three different *C. difficile* detection methods using fecal specimens from patients with diarrhoea, and the clinical applicability of the three different detection methods was compared. The purpose of this study is to provide a laboratory basis for the diagnosis, treatment, prevention, and control of *C. difficile* infection-related diseases.

## 2. Methods

### 2.1. Stool Samples

This study was conducted in accordance with the clinical practice guidelines for *Clostridium difficile* infection (CDI) in adults and children updated by the Infectious Diseases Society of America (IDSA) and the Society for Healthcare Epidemiology of America (SHEA) in 2017 [6]. A total of 150 stool specimens were collected from patients over 2 years of age who developed diarrhoea (3 or more stools during a 24 hour period) from December 2016 to August 2018 at the Shengjing Hospital of China Medical University. The average age of the patients was 55.3 years, and 44.0% (66) were males and 56.0% (84) were females. The *C. difficile* positive strains (BI/NAP1/027) were donated from the Peking Union Medical College Hospital serving as the positive control of the study.

### 2.2. Toxigenic Culture (TC)

The stool sample was inoculated onto CDIF agar (bioMérieux, France). Simultaneously, a stool specimen (1.0 mL) was mixed with an equal volume of anhydrous ethanol and incubated for 1 hour at room temperature. Then, the mixed stool sample was inoculated onto anaerobic blood agar (JIZHANG Limited) and CCFA agar (Oxoid Limited). The agars were incubated at 37°C for at least 24 hours before final interpretation of the results. When positive culture results were obtained, the isolated *C. difficile* colonies were tested for toxin production using a multiplex PCR-based toxin gene test (BBI Life Science Corporation, 01035, HK).

The multiplex amplification of toxins was performed as described previously [7], and the primers used are shown in Table 1. Bacterial colonies were prepared for PCR as follows: 20–30 colonies were transferred into 200 *μ*L of 10.0% Chelex-100 (Solarbio Ltd) in TE (10 mM Tris-HCl, 1 mM EDTA, pH 8), boiled for 10 minutes, and then centrifuged briefly. The supernatant was then used for PCR. A 5-plex PCR was developed for the detection of tcdA, tcdB, cdtA, cdtB, and 16S rDNA. The PCR reactions were run in total volumes of 50 *μ*L containing the following reagents: 27 *μ*l Taq PCR Master Mix, 1 *μ*l DNA template, 9.5 *μ*l PCR water, and 12.5 *μ*l primer mixture. The thermocycler conditions were 10 min at 94°C, followed by 35 cycles of 50 seconds at 94°C, 40 seconds at 56°C, and 50 seconds at 72°C, and a final step of 3 minutes at 72°C.

### 2.3. Enzyme Immunoassays (EIA)

All specimens were tested simultaneously for GDH (Glutamate dehydrogenase, a conserved antigen which is abundantly present on the surface of both toxigenic and nontoxigenic strains of *C. difficile*) and A/B toxins using commercial VIDAS kits (bioMérieux, Marcy-l'Étoile, France) according to manufacturer's instructions. For the GDH assay, a negative result was defined as optical density (OD) 450/630 nm < 0.10, and a positive result was defined as OD 450/630 nm ≥ 0.10. The fluorescence intensities for A/B toxin of <0.13, ≥0.13 to <0.37, and ≥0.37 were considered negative, equivocal, and positive, respectively.

### 2.4. The GeneXpert *C. difficile* PCR Assay

The GeneXpert *C. difficile* PCR assay, a real-time PCR assay, was carried out in accordance with the manufacturer's instructions. The specimen was vortexed at high speed for 15 seconds, and a sterile dry swab was then dipped into the stool for testing. The excess stool was removed, and the swab was placed into a vial containing the sample reagent. The swab's stem was then broken off after lifting it a few mm, so that the cap could be closed tightly. The vial was then vortexed at high speed for 10 seconds. All the liquid from the sample was transferred into the “S” chamber of the cartridge using a large transfer pipette (Cepheid), and the chamber was then placed into the GeneXpert Dx System instrument for analysis. The results were determined by the GeneXpert Dx System using measured fluorescence signals and embedded algorithms.

## 3. Results

### 3.1. Detection of *C. difficile* Toxin-Positive Strains

In fecal specimens from 150 patients with diarrhea, 26 toxin-positive strains were detected by multiplex PCR, and both A and B toxins were present. No binary toxin-positive strains were found. Overall, 17.33% of the cases tested were toxin-positive (26/150). Thirty-seven positive samples were detected by the VIDAS GDH assay, and 15 positive samples were detected by the VIDAS toxin A/B assay. Seventy-nine specimens were tested using the GeneXpert *C. difficile* PCR assay, and 18 toxin B-positive cases were detected.

### 3.2. Comparison of Initial Screening Methods for CDI

To compare the sensitivity and specificity of the CDI detection methods in question, the anaerobic culture method was used as a reference method. First, the clinical application of the VIDAS *C. difficile* GDH assay was evaluated. Compared with the culture method, the sensitivity, specificity, positive predictive value (PPV), and negative predictive value (NPV) of the VIDAS GDH assay were 100.0%, 97.4%, 91.9%, and 100.0%, respectively. The sensitivity and NPV of the VIDAS GDH assay were both 100.0%, and consistency analysis revealed that the results of the VIDAS assay and the culture method were highly consistent (Kappa = 0.945). A chi-square test showed that there was no statistical difference between the two methods (*χ*^2^=1.33,  *P* > 0.05) (Table 2).

### 3.3. Comparison of *C. difficile* Toxin Detection Methods

The toxigenic culture method was next used as a reference to evaluate the clinical application of the GeneXpert *C. difficile* PCR assay and the VIDAS enzyme immunoassay. The sensitivity, specificity, PPV, and NPV of the GeneXpert *C. difficile* PCR assay were 100.0%, 96.8%, 88.9%, and 100.0%, respectively, and the sensitivity, specificity, PPV, and NPV of the VIDAS enzyme immunoassay were 55.6%, 100.0%, 100.0%, and 88.4%, respectively. The results showed that there was no significant difference between the GeneXpert *C. difficile* PCR assay and the reference method (*χ*^2^=0.50,  *P* > 0.05) (Table 3). The results also showed no statistically significant differences between the VIDAS enzyme immunoassay and the reference method (*χ*^2^=3.125,  *P* > 0.05) (Table 4). The diagnostic parameters of the GeneXpert *C. difficile* PCR assay and the VIDAS enzyme immunoassay results are shown in Table 5. Compared to the reference method, the GeneXpert *C. difficile* PCR assay has high sensitivity and specificity. The VIDAS enzyme immunoassay has a high specificity (100.0%), but the sensitivity is only 55.6%. Consistency analysis showed that the consistency between the GeneXpert assay and the reference method was significantly higher (Kappa = 0.925) than that of the VIDAS enzyme immunoassay and the reference method (Kappa = 0.659).

**Figure 1:**
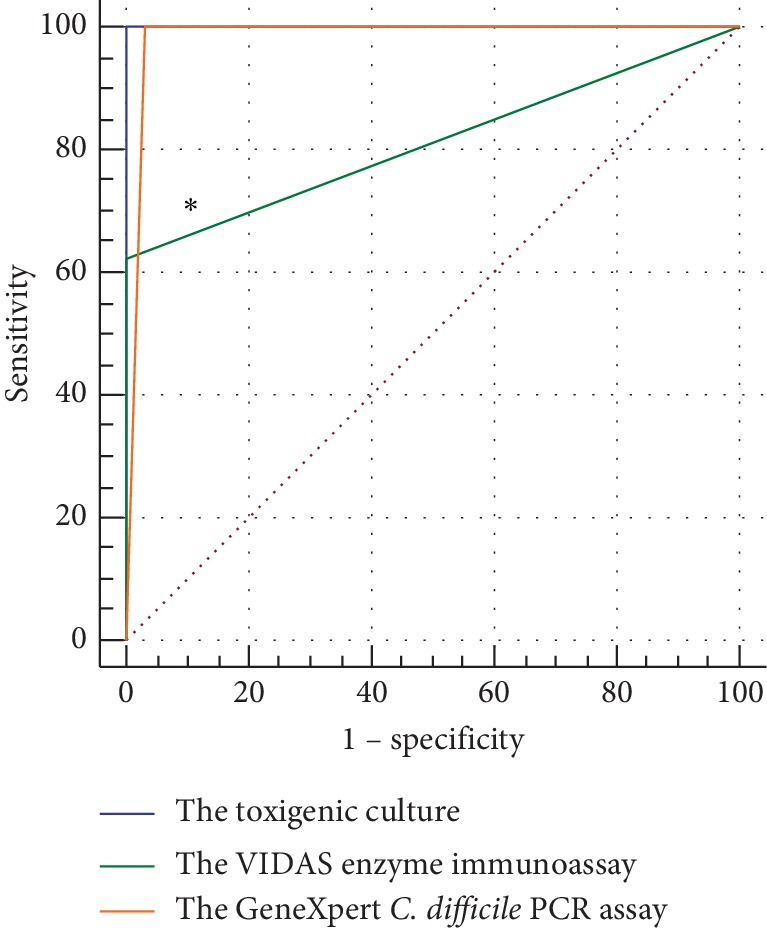
The area under the ROC curve of the toxigenic culture, the GeneXpert *C. difficile* PCR assay, and the VIDAS enzyme immunoassay (79 specimens). The area under the GeneXpert *C. difficile* PCR assay and the VIDAS enzyme immunoassay curves were 0.984 and 0.813, respectively. ^*∗*^Significantly different as compared with the toxigenic culture method (*P<0.05*).

The area under the ROC curve of the three methods was compared and statistically tested. The area under the GeneXpert *C. difficile* PCR assay and the VIDAS enzyme immunoassay curves were 0.984 and 0.813, respectively. There was a significant difference between the area under the curve of the VIDAS enzyme immunoassay and the toxigenic culture method (*Z*=3.000, *P* < 0.05), and there was no significant difference between the area under the curve of the GeneXpert *C. difficile* PCR assay and the toxigenic culture method (*Z*=1.426, *P* > 0.05). The results are summarized in Table 6 and Figure 1.

## 4. Discussion

*C. difficile* is a major cause of antibiotic-associated diarrhoea. The methods commonly used for *C. difficile* detection include culture methods, enzyme immunoassays, and molecular biology methods [8]. At the present time, only some areas of China carry out routine detection of *C. difficile* in patient samples. A meta-analysis published in 2016 suggested that the incidence of toxigenic CDI with diarrhoea in China from 2010 to 2016 was 14.0% [9]. In the same year, another published meta-analysis suggested that the pooled incidence rate of CDI was 19.0% [10]. In our study, the rate of detection of toxigenic *C. difficile* was 17.3%, similar to those previously reported.

For the initial screening methods for CDI, the anaerobic culture method is routinely used to detect CDI. However, this method takes 1–3 days to complete, which is not conducive to rapid clinical diagnosis [5]. Additionally, this method cannot distinguish whether the strain is toxigenic or not. The greatest advantage of the GDH enzyme immunoassay is that it has a high sensitivity and NPV [11]. In this study, the performance of the VIDAS GDH assay was evaluated in comparison to the culture method. The results showed that there was no statistically significant difference between the two methods. The sensitivity and NPV of the VIDAS GDH method reached 100.0%, which was consistent with previous reports [12–15]. These results indicate that the VIDAS GDH assay is a reliable initial screening test for CDI, and it may be used as an initial test in two- or three-step algorithms for CDI diagnosis. Therefore, further tests may not be necessary in GDH-negative samples. The VIDAS GDH assay is quick and relatively inexpensive to perform. However, this method cannot distinguish between toxin-positive and toxin-negative strains [16]. Since the detection of toxins is necessary for CDI diagnosis, this assay cannot be used independently to diagnose CDI.

Toxigenic culture was also used as a reference method to evaluate the performance of the GeneXpert *C. difficile* PCR assay and the VIDAS enzyme immunoassay. The results showed no statistically significant differences between the GeneXpert *C. difficile* assay and the toxigenic culture method. The results also showed no significant differences between the VIDAS enzyme immunoassay and the toxigenic culture method. The GeneXpert *C. difficile* PCR assay had high sensitivity and specificity, while the VIDAS enzyme immunoassay had high specificity but low sensitivity. The experimental data show that the sensitivity of the toxin enzyme immunoassay varies greatly (from 40.0% to 100.0%) [17–20] There are a few factors which may contribute to this: first of all, the toxigenic culture method cannot quantitatively detect *C. difficile*. It can only detect whether toxigenic *C. difficile* is present in the feces. The VIDAS enzyme immunoassay can quantitatively detect *C. difficile* toxin protein, but if the amount of *C. difficile* in the patient sample is less than the lower limit of detection, a false negative result is possible. At the same time, the VIDAS toxin A/B assay is limited by the time of detection. If the specimen is in transit for too long or improperly transported, the toxin proteins in the stool specimen may degrade, which may also lead to false negative results.

The toxigenic culture method is the gold standard for detection of *C. difficile*. However, the cultivation time is long (2-3 days), and it is cumbersome to perform [11,15,21]. The VIDAS enzyme immunoassay is easy to perform, and the results are available in 2-3 hours, but false negative results can occur if the toxin proteins are allowed to degrade. The GeneXpert *C. difficile* assay combines sample purification, nucleic acid amplification, and target sequence determination in simple or complex samples to report results in a short period of time. It is fast, accurate, and easy to use, but it is also very expensive [22].

One limitation of this study is that due to financial reasons, only the first 79 specimens were tested using the GeneXpert *C. difficile* assay. The sensitivity, specificity, PPV, and NPV of the VIDAS enzyme immunoassay using all 150 specimens were 50.0%, 99.2%, 92.9%, and 90.4%, respectively. The sensitivity of the VIDAS enzyme immunoassay using 150 specimens was 5.6% lower than the results obtained when using only the first 79 specimens. However, both results suggest that the VIDAS enzyme immunoassay has low sensitivity. Since only 79 specimens were used to compare the sensitivities, the random sampling error may be inflated. Simultaneously, the small sample size may affect the analysis of other potential clinical differences between patients, such as analysis of risk factors for *C. difficile* infection. In our study, samples were collected for almost 2 years, and the results showed that the prevalence of *C. difficile* infection was consistent with that reported domestically. This may be attributable to a lack of awareness on the part of clinicians concerning the clinical diagnosis of the disease.

Future clinical studies using the GeneXpert PCR assay to detect *C. difficile* should focus on the false positives. A larger sample size should be analyzed, and patient follow-up should occur in order to further evaluate the efficiency of this method.

The prevalence of *C. difficile* may be underestimated because doctors and patients know little about the disease. Therefore, increasing publicity and educating clinicians and patients about the disease should be a high priority. The *C. difficile* spores can survive in the environment for several months and can resist high temperatures, oxygen, and disinfectants; this may play a role in the transmission of *C. difficile* [23]. A recently published study examined wards of patients infected with *C. difficile* and concluded that increased sample surface area in the ward was related to increased numbers of recovered spores and increased proportions of positive samples. This study also found that *C. difficile* contamination was common on hospital railings and floors, especially in the rooms of patients with *C. difficile* infection [24]. Further studies should focus on the transmission of *C. difficile*, especially in hospital settings.

Presently, no flawless methods of detection for *C. difficile* exist, and all of the current methods have shortcomings. Future studies should focus on the regulation of spore formation and toxin genes, which may enable development of a new detection method. Discovery and detection of genes that regulate *C. difficile* toxin and spores, combined with the measurement of *C. difficile* toxin and spore gene levels, may enable rapid detection of *C. difficile*. Simultaneously, it may help guide the diagnosis, treatment, and evaluation of *C. difficile* infection after treatment.

In conclusion, the VIDAS GDH assay was found to be useful as an initial screening method due to its excellent sensitivity and NPV. The VIDAS enzyme immunoassays are affected by factors such as detection time, and are therefore more prone to false negative results. The GeneXpert *C. difficile* PCR assay is simple, quick, accurate, and valuable for clinical detection of *C. difficile*.

## Figures and Tables

**Table 1:** Primers used in the present analysis.

Gene target	Primer name	Sequence (5′ to 3′)	Primer concentration (*μ*M)	Amplicon size (bp)
tcdA	tcdA-F3345	GCATGATAAGGCAACTTCAGTGGTA	0.6	629
	tcdA-R3969	AGTTCCTCCTGCTCCATCAAATG	0.6	

tcdB	tcdB-F5670	CCAAARTGGAGTGTTACAAACAGGTG	0.4	410
	tcdB-R6079A	GCATTTCTCCATTCTCAGCAAAGTA	0.2	
	tcdB-R6079B	GCATTTCTCCGTTTTCAGCAAAGTA	0.2	

cdtA	cdtA-F739A	GGGAAGCACTATATTAAAGCAGAAGC	0.05	221
	cdtA-F739B	GGGAAACATTATATTAAAGCAGAAGC	0.05	
	cdtA-R958	CTGGGTTAGGATTATTTACTGGACCA	0.1	

ctdB	ctdB-F617	TTGACCCAAAGTTGATGTCTGATTG	0.1	262
	cdtB-R878	CGGATCTCTTGCTTCAGTCTTTATAG	0.1	

16S rDNA	PS13	GGAGGCAGCAGTGGGGAATA	0.05	1062
	PS14	TGACGGGCGGTGTGTACAAG	0.05	

**Table 2:** Comparison of the VIDAS *C. difficile* GDH assay and the anaerobic culture method.

The VIDAS GDH assay	The anaerobic culture method		Total
	Positive	Negative	
Positive	34	3	37
Negative	0	113	113
Total	34	116	150

**Table 3:** Comparison of the GeneXpert *C. difficile* PCR assay and the toxigenic culture method.

The GeneXpert *C. difficile* PCR assay	The toxigenic culture method		Total
	Positive	Negative	
Positive	16	2	18
Negative	0	61	61
Total	16	63	79

**Table 4:** Comparison of the VIDAS enzyme immunoassay and the toxigenic culture method.

The VIDAS enzyme immunoassay	The toxigenic culture method		Total
	Positive	Negative	
Positive	10	0	10
Negative	8	61	69
Total	18	61	79

**Table 5:** Diagnostic parameters of laboratory test methods.

Method	Sensitivity	Specificity	PPV	NPV	Kappa value
The GeneXpert *C. difficile* PCR assay	1.000	0.968	0.889	1.000	0.925
The VIDAS enzyme immunoassay	0.556	1.000	1.000	0.884	0.659

**Table 6:** Comparison of the area under the ROC curve between the toxin detection methods of 79 specimens.

Method	Z value	*P* value	Area under the curve
The toxigenic culture	1.426	0.1539	1.000
The GeneXpert *C. difficile* PCR assay			0.984

The toxigenic culture	3.000	0.0027	1.000
The VIDAS enzyme immunoassay			0.813

## Data Availability

The data used to support the findings of this study are included within the article.
